# Physical Activity Associated with Public Transport Use—A Review and Modelling of Potential Benefits 

**DOI:** 10.3390/ijerph9072454

**Published:** 2012-07-12

**Authors:** Chris Rissel, Nada Curac, Mark Greenaway, Adrian Bauman

**Affiliations:** Prevention Research Collaboration, The University of Sydney, 92-94 Parramatta Road, Camperdown, NSW 2050, Australia; Emails: n.curac@sydney.edu.au (N.C.); mgree@doh.health.nsw.gov.au (M.G.); adrian.bauman@sydney.edu.au (A.B.)

**Keywords:** public transport, mass transit, walking, physical activity

## Abstract

Active travel, particularly walking and cycling, has been recommended because of the health benefits associated with increased physical activity. Use of public transport generally involves some walking to bus stops or train stations. This paper is a systematic review of how much time is spent in physical activity among adults using public transport. It also explores the potential effect on the population level of physical activity if inactive adults in NSW, Australia, increased their walking through increased use of public transport. Of 1,733 articles, 27 met the search criteria, and nine reported on absolute measures of physical activity associated with public transport. A further 18 papers reported on factors associated with physical activity as part of public transport use. A range of 8–33 additional minutes of walking was identified from this systematic search as being attributable to public transport use. Using “bootstrapping” statistical modelling, if 20% of all inactive adults increased their walking by only 16 minutes a day for five days a week, we predict there would be a substantial 6.97% increase in the proportion of the adult population considered “sufficiently active”. More minutes walked per day, or a greater uptake of public transport by inactive adults would likely lead to significantly greater increases in the adult population considered sufficiently active.

## 1. Introduction

Promoting physical activity is an established health promotion priority, and is as important stopping smoking and reducing high blood pressure [1]. It is well documented that population patterns of physical activity are influenced by the physical and built environments [2,3] with features of the built environment such as mixed land use, well-connected street networks and high residential density positively associated with higher levels of physical activity [4,5,6]. 

Single mode walking or cycling trips are generally the focus of research examining the effects of active transport on health, and often this is in the context of the journey to work [7,8,9]. However this may exclude the walking or cycling component of a trip that is mostly a public transport trip, given that the greatest time or distance is spent on public transport for that journey. The beginning or end of a public transport trip usually involves some walking to the next destination. This active travel component of a public transport trip could provide an important opportunity for physical activity [10] and may be missed in some assessments of physical activity.

To date there have been no systematic reviews of the literature examining physical activity associated with public transport use. The purpose of this paper is to systematically examine the extent of association between the use of public transport and time spent in physical activity (walking/cycling to transport stops/stations) among adults. In addition using statistical modelling we examine the potential effect on the population level of physical activity if inactive adults in NSW, Australia, were to increase their walking by the amount found to be attributable to public transport in this review.

## 2. Methods

### 2.1. Study Selection Criteria

Study inclusion criteria were any papers reporting on the relationship between public transport use and physical activity levels in adults published in the last ten years (2002–2012). All modes of public transport were included (for example, trams, trains, light rail, ferries, buses), but not single mode walking, cycling, freight transport and taxi trips. Walking and cycling to and from public transport stations were included. The review included all types of study design. Papers which reported on the relationship between public transport use and health status (for example, obesity, BMI) were also included. Excluded from the review were papers focused on land use planning and changes to the built environment aimed at facilitating physical activity which did not specifically include data on the extent of physical activity associated with public transport use. Articles generally discussing the issue or tangentially related topics were also excluded. 

### 2.2. Search Strategy and Study Selection Process

**Figure 1:**
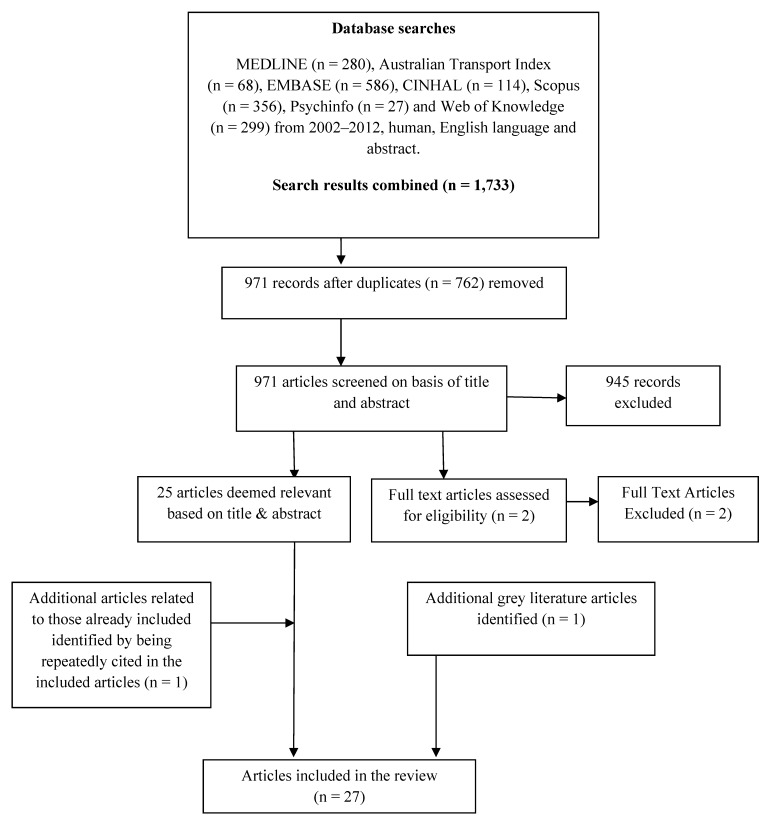
Summary of Search Strategy and Identification of Articles Included in the Review.

Studies were identified through searching of the following electronic databases (January 2002–2012): Medline, Australian Transport index, Embase, Cinahl, Scopus, Psychinfo and Web of Knowledge limited to humans, English language and abstract. Grey literature (including government and agency reports) was included where it was frequently cited by other papers, or was readily identified through a Google search. The search focused on three key elements: (1) Population (adults); (2) physical activity and (3) public transport. Key search terms are listed in Table 1. These terms were mapped to appropriate subject headings and searched as a keyword in each database. All articles were imported into an Endnote library and duplicates removed (see Figure 1). 

An initial screen of titles and abstracts was undertaken by one researcher (NC) to identify articles meeting the study inclusion criteria. The full text of potentially relevant articles not clearly identified from the title/abstract were obtained and assessed for eligibility. The set of 29 possible articles for inclusion were reviewed by a second author (CR), which led to two being excluded. 

**Table 1:** Electronic search strategy.

Key words	Medline/Psychinfo terms	Australian Transport Index	Embase	Scopus	Cinahl	Web of Knowledge
**Adult**	Adult$	Adult$	Adult$	Adult$	Adult$	Adult$
**Physical Activity**	Physic$ activ$, exercis$, physical training, fitness training	No relevant subject term	Physic$ activ$, exercis$, physical training, fitness training	Physic$ activ$, exercis$, physical training, fitness training	Physic$ activ$, exercis$, physical training, fitness training	Physic$ activ$, exercis$, physical training, fitness training
	Physical fitness	No relevant subject term	Physical fitness	Physical fitness	Physical fitness	Physical fitness
	Physical exertion	No relevant subject term	No relevant subject term	Physical exertion	No relevant subject term	Physical exertion
	Walk$, active travel, active commut$, active transport$	Walk$, active travel, active commut$, active transport$	Walk$, active travel, active commut$, active transport$	Walk$, active travel, active commut$, active transport$	Walk$, active travel, active commut$, active transport$	Walk$, active travel, active commut$, active transport$
	Bicyc$, cycle, cycling, biking	Bicyc$, cycle, cycling, biking	Bicyc$, cycle, cycling, biking	Bicyc$, cycle, cycling, biking	Bicyc$, cycle, cycling, biking	Bicyc$, cycle, cycling, biking
	Leisure activ$	No relevant subject term	No relevant subject term	Leisure activ$	Leisure activ$	Leisure activ$
**Public Transport**	Public transport$, public transit$, rail$, tram, metro, bus, ferry, subway, mass transit.	Public transport$, public transit$, rail$, tram, metro, bus, ferry, subway, mass transit.	Public transport$, public transit$, rail$, tram, metro, bus, ferry, subway, mass transit.	Public transport$, public transit$, rail$, tram, metro, bus, ferry, subway, mass transit.	Public transport$, public transit$, rail$, tram, metro, bus, ferry, subway, mass transit.	Public transport$, public transit$, rail$, tram, metro, bus, ferry, subway, mass transit.

Key search terms, mapped to appropriate subject headings in each database and searched as a key word in all databases. *MeSH: Medical subject heading (Medline medical index term); the dollar sign ($) stands for any character(s).* All searches limited to English, humans, abstract and 2002–2012.

### 2.3. Modelling of NSW Health Survey Data

We also sought to explore the likely impact on the population of NSW in terms of the overall proportion of the adult population considered sufficiently active (defined as meeting the global physical activity recommendations of “150 or more minutes of at least moderate intensity physical activity” during the week) if there were increases in physical activity associated with increased public transport use. Data were drawn from the NSW Continuous Health Survey conducted in 2010 [11]. The NSW Continuous Health Survey is conducted by telephone among a representative sample of residents aged 16 years or over in NSW, Australia. The variable of interest was minutes of physical activity per week. 

The distribution of physical activity in the inactive population and total population was modelled based on three scenarios where there was an increase in 8, 16, or 24 minutes of physical activity per week (five days, to represent using public transport during a working week), and if 10, 20 or 30% of the adult population added these additional minutes. All statistical analysis was done using the software package “R” [12]. All statistical analyses were weighted using the NSW Health Survey post-stratification weights. Minutes of physical activity per week was estimated using the weighted empirical distribution function. To calculate 95% confidence intervals, a survey bootstrapping technique was used with 1,000 replicates and the 2.5% and 97.5% quintiles of the replicates were found [13].

Percentage improvement in the proportion of the population who were sufficiently active as a function of additional minutes of physical activity per week was estimated using the weighted cumulative density function. The graph of this function was then smoothed using splines of the maximum degree which still retained the convexity of the functions [14]. 

## 3. Results

Using the search strategy described above, 1,733 articles were generated (see Figure 1). After removal of duplicates (n = 762), 971 article titles and abstracts were screened for relevance, with 27 articles meeting the eligibility criteria. Table 2 summarises nine studies where physical activity in relation to public transport was measured in absolute terms (using accelerometers or pedometers). Six were from the USA, two from the UK and one from Australia. None addressed cycling. While not using the same measurement units for physical activity, it appears that there are at least 8 minutes of additional physical activity [15] associated with public transport use a day, and several studies reported a range up to 12–15 minutes a day [15,16,17]. One study found public transport users accumulated up to about 24 minutes of walking a day, but did not examine walking related to car use [18]. The Australian study [17] was consistent with the USA and UK studies (and was in the 12–15 minutes of walking range), strengthening the likelihood that the overseas data is relevant to the Australian context. The median walking time associated with public transport use was 15 minutes. 

**Table 2:** Studies reporting objectively measured physical activity in adults in relation to public transport use.

Study Characteristics		Methodology					Outcome	
Author, Year, Country,	Research Question	Public Transport Measure		Physical Activity Measure	Occupational/Leisure-Time Physical Activity Separated in Final Analyses	Confounders Measured	Result	
Study Design,								
Sample Size/Demographic								
Lachapelle, U *et al*. (2011) [15]	Relationship between commuting by public transport and objectively measured moderate intensity physical activity.	Reported % of all work commute trips taken by public transport. 3 groups:		Mean daily minutes of accelerometer measured moderate intensity physical activity.	Self report measures of occupational/ leisure-time physical activity did not confound results.	(1) Neighbourhood walkability, enjoyment of physical activity, demographics.	(1) Frequent public transport users accumulated significantly more (+8 mins) moderate-intensity physical activity daily compared with non-public transport users.	
USA								
Cross-sectional		-non public transport user						
n = 1,237		-infrequent public transport user(<50% commutes by public transport)						
20–65 years old working outside home		-frequent public transport user(≥50% commutes by public transport)						
Besser, LM *et al*. (2005) [18]	Estimate the daily level of physical activity obtained by Americans solely by walking to/from transit.	Only measured walking in transit users so no public transport measure.		Minutes spent walking to/from transit in a 24 h period.	Only walking to/from transit measured.	(1) Uncontrolled	(1) People who walk to/from transit accumulate 24.3 mins of mean walking time/day.	
USA								
Cross-sectional								
n = 3,312								
18+ years who walked to/from transit on day of measurement						(2) Stratified for transit type, demographics, population density, car ownership.	(2) 29% of transit users achieve ≥ 30 minutes walking to/from transit daily.	
Edwards, R *et al*. (2008) [16]	Is the additional walking associated with mass transit use large enough to reduce obesity & health care costs? (by estimating additional walking associated with public transport use).	“Public transit user” = anyone who reports using public transport for any reason on assigned travel day.		Time spent walking on assigned travel day for any purpose.	no	(1) Demographics, number of household vehicles, own home, census region fixed effects.	(1) Public transport use associated with significantly more (8–10 mins) additional walking per day.	
USA								
Cross-sectional								
n = 28,771								
18+ years old from National Household travel survey								
Evans, A *et al*. (2009) [19]	Focus is on rail and road safety.	Only examined walking in rail users so no public transport measure.		Self report distance walked to surface railway stations over 7 consecutive reporting days.	Only measured walking to train stations.	no	Brits walk an average of 0.905 km per journey on journeys with surface rail as the main mode.	
UK								
Cross-sectional								
n = 5,749 rail journeys								
Data from British National Travel survey 1999–2001							(equivalent to 10–12 minutes per trip)	
Morabia *et al*. (2010) [20]	Compare levels of physical activity between car & public transport commutes to work.	18 participants commuted by car to Queens College for 5 days than switched to commuting by public transport. (no public transport measure)	Activity diary +GPS system used to calculate the average metabolic equivalent value for car *vs*. public transport.		n/a as experiment limited to walking for transport.	no	Public transport commuters expended significantly more (+622 kcal over 5 days) energy compared with travelling the same route by car. (approximately equivalent to 30 minutes walking)	
USA								
Experimental								
n = 18								
Adults either working/studying at Queens College								
Wener, R *et al*. (2007) [21] .	Compare level of physical activity between car and transit users travelling to/from work.	If travelled to work by:	Pedometer worn for 5 days and international physical activity questionnaire issued at start of measuring week.		no	(1) Income, gender & education.	(1) Train commuters walked significantly more steps (2,000 per day) compared to car commuters (equivalent to about 30 minutes).	
USA								
Cross-sectional								
n = 177								
Adults commuting from New Jersey to work in NY		public transport ≥ 4 × /week = transit user car ≥ 4 × /week = car user.				(2) Income, gender, education & commuting time.	(2) Train commuters 4 × more likely to achieve 10,000 steps/day compared to car users.	
Davis, M *et al*. (2011) [22]	Describe the frequency, purpose & travel mode of daily trips in older adults & their association with participant characteristics & objectively measured physical activity.	Determined by respondent noting “mode of transport” in trip log.	Steps/day and minutes of moderate-intensity physical activity day assessed by accelerometer for 1week + daily trip log noting purpose of trip/mode of transport .		Did not adjust for “purpose of trip”.	(1) Other trip types (car, walking, cycling), age, sex, physical function, use of a walking aid, education & car ownership.	(1) Each weekly trip made by public transport is significantly associated with extra 412.7 steps/day in older adults (equivalent to about 8 minutes of walking).	
UK								
Cross-sectional								
n = 214								
Adults over 70 years old						(2) As per #1	(2) Public transport trips made by older adults is significantly associated with minutes of moderate-intensity physical activity/day (ln = 0.06).	
Villanueva, K *et al*. (2008) [17]	Compare pedometer-determined physical activity levels of university students using public transport compared to cars for travel to uni.	Categorised into 2 groups: “mainly car user” or “mainly public transport user” for travel to uni.	Time spent walking for transport estimated from pedometer & diaries.		Adjusted for self-report leisure-time physical activity in analysis #2.	(1) Uncontrolled	(1) Public transport users took significantly greater steps (11,433 *vs* . 10,242) compared with drivers.(1,191 steps is equivalent to about 15 minutes of walking)
Australia (Perth)							
Cross-sectional							
n = 103							
University students						(2) Gender, age and leisure-time physical activity.	(2) Public transport users significantly (3.55×) more likely to achieve 10,000 steps/ day compared with drivers.
Macdonald, J *et al*. (2010) [23]	Examine association between objective & perceived measures of the built environment, body mass index, obesity and meeting recommended physical activity (RPA) through walking and vigorous exercise. To assess effect of using light rail on weekly RPA.	Pre and post exposure to a new light rail transit line.	Categorised as either meeting the recommendations for physical activity through vigorous exercise or moderate-intensity physical activity (through walking) or not meeting recommendations.		no	(1) Age, gender, race, employment status, education, own residence, distance to work, perception of neighbourhood, access to parks, density of food/alcohol establishments, household density, use of public transport on weekly basis & propensity to use light rail.	(1) Light rail transit (LRT) users decreased their body mass index by average of 1.18 compared with similarly situated non-LRT users over 12–15 months follow-up.
USA							
n = 498							(2) LRT users lived 1.5 miles from stations (equivalent to 36 minutes walking).
Cross-sectional and pre/post intervention						2) As per #1	(3) Association between LRT use and meeting weekly recommended physical activity levels by walking was in a positive direction but not significant.

In the US, 29 percent of those who use transit were physically active for 30 minutes or more each day (and considered as sufficiently active), solely by walking to and from public transit stops [18]. A similar result was found in the Australian context, with public transport users 3.5 times more likely to meet the recommended step target of 10,000 steps compared with car drivers [17]. Similarly in the US, transit users took 30 percent more steps per day and spent 8.3 more minutes walking per day than did people who relied on cars [16]. For seniors, each public transport trip in the UK was associated with an extra 412 steps, equivalent to about 8 minutes of walking (allowing for a slower speed) [22].

An Australian report (using Victorian Travel Survey data) reported that people who used public transport on a particular day also spent an average 41 minutes walking and/or cycling as part of their travel. This is five times more physical activity than those who only use private transport, who on average only spend 8 minutes walking or cycling for transport, and representing an additional 33 minutes of physical activity [10]. Public transport users (*i.e.*, subways, light or heavy rail, buses, trolleys, or ferries) were less likely to be sedentary or obese than adults who did not use public transport [24]. Conversely, motor vehicle travel was associated with higher obesity rates at both the county and individual level [8,24,25] (there are a range of benefits associated with public transport use, and the 18 papers reporting these findings are summarised in the Appendix).

These studies measured walking in some way, but did not always differentiate between single mode walking for transport (which was excluded from this review) or walking to public transport (multi-mode). Some of the papers reported total minutes walking for transport, and factors associated with it. Compared with motor vehicle use, there were clear health benefits for users of public transport, particularly lower weight. A number of the papers compared energy expenditure of car users compared to public transport users, and all have concluded that there is significantly greater energy expenditure for public transport users [8,20,24].

There were higher levels of walking when public transport access points (stations and bus stops) were closer, and these associations were generally significant [25,26,27,28,29,30,31,32,33,34]. Good access to public transport is significantly associated with walking sufficiently per week to meet physical activity recommendations. Of the two longitudinal papers one showed significant increases in physical activity associated with new public transport stops/stations [28] but the other did not, although it reported that limited public transport availability was associated with low transport walking [29].

### 3.1. Modelling of NSW Health Survey Population Data

Using NSW Continuous Health Survey data for adults, almost half of women (49.8%) and 60.7 percent of men are classified as sufficiently physically active [35]. There is a clear dose-response relationship between the proportion of the population achieving 150 minutes per week of physical activity (and considered “sufficiently physically active”) by the addition of either 8, 16 or 24 minutes of walking. Conservatively, if only 20% of inactive people in NSW walked for 16 minutes more each week, across the state there would be 6.97% more adults meeting public health recommendations for physical activity, which has significant public health implications (see Table 3). As very few public health interventions increase population physical activity by anything like this amount, this would represent a significant improvement.

**Table 3:** Population increase in the proportion of NSW adults who are sufficiently physically active by increases in minutes of physical activity and the percent uptake by those currently inactive.

		Minutes of physical activity added per weekday		
		8	16	24
**Percent uptake of physical activity by insufficiently active**	**10%/**	1.96%	3.48%	5.94%
		(1.81%–2.12%)	(3.36%–3.61%)	(5.62%–6.26%)
	**20%**	3.93%	6.97%	11.88%
		(3.62%–4.23%)	(6.61%–7.32%)	(11.45%–12.31%)
	**30%**	5.89%	10.45%	17.82%
		(5.10%–6.68%)	(10.00%–10.94%)	(17.00%–18.64%)

**Figure 2:**
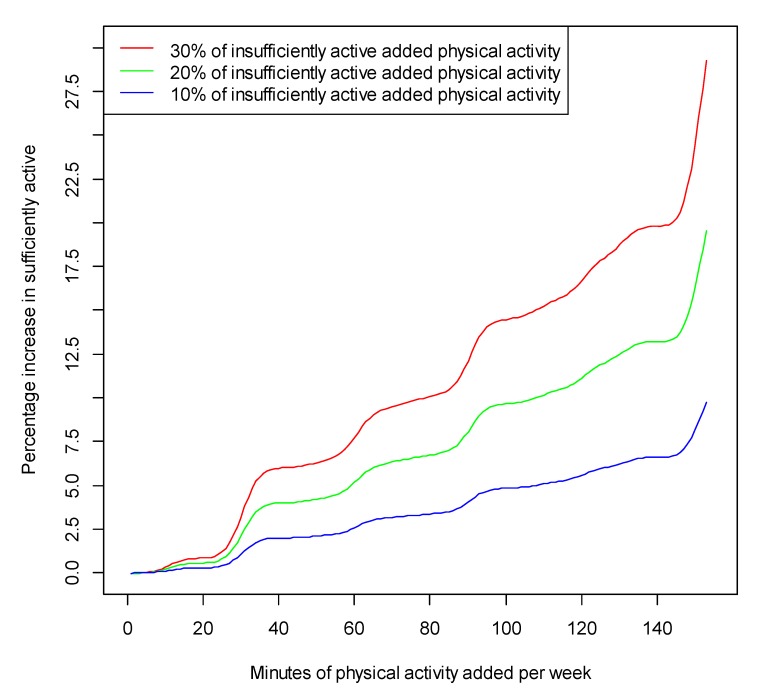
Proportion of the NSW population considered sufficiently active by increases in minutes of physical activity per week associated with three scenarios of take up of public transport among insufficiently active NSW residents.

This relationship is illustrated in Figure 2, which shows the increase in the proportion of the NSW population considered sufficiently active by increases in minutes of physical activity per week hypothetically associated with three scenarios of take up of public transport among insufficiently active NSW residents. 

## 4. Discussion

There is relatively little data available on the extent of physical activity associated with public transport use, but it is clear that there is an additional amount ranging from 8 to 33 minutes of walking per day. The Australian Government recommends that adults should get at least 30 minutes of moderate intensity physical activity on most, preferably all, days of the week [36]. The globally accepted cut-point for sufficient physical activity for health is 150 minutes of moderate-intensity physical activity per week [37]. For some people transport related walking is sufficient to achieve the recommended levels of physical activity. Our statistical modelling shows that increases in people walking for transport, by as little as 8 minutes a day, would lead to significant increases in physical activity and improved population health in NSW.

There is much more data available on the health benefits of a modal shift away from motor vehicles to active travel (including public transport). In general, policy initiatives that favour active travel have many co-benefits [38] and even in the absence of complete data, there are many benefits from such a shift, including less congestion, less air and noise pollution, and stronger sense of local community. 

### 4.1. Study Strengths and Limitations

The current review has a number of strengths and weaknesses. Strengths include the original nature of the review focusing on objective measures of physical activity in relation to public transport use with minutes of walking typically calculated from accelerometers, pedometers or travel diaries, rather than self-report. Also original is the population modelling of the likely effect of increased transport use by inactive adults using population survey data for NSW. 

A limitation of the study was the review only covered the last ten years. However, this period included most of the known research on this topic. Another limitation is that only one of the researchers screened the initially identified articles, and this may have led to the exclusion of relevant papers. A third limitation is the large variation of measures used to assess physical activity and energy expenditure, and that the data come from a range of study types.

## 5. Conclusions

The nine papers identified in this review report a range of 8–33 minutes of additional physical activity associated with public transport use, with several papers reporting 12–15 minutes. Using bootstrapping analyses, we found that if public transport use by inactive adults was to increase, there would be a significant dose-response increase in the population level of sufficiently active adults in NSW.

## Appendix

**Table 4:** Studies reporting associations between the use of public transport and health outcomes in adults.

Study Characteristics		Methodological					Outcome	
Author, Year, Country,Study Design, Sample Size/Demographic	Research Question	Public Transport Measure	Physical Activity Measure	Occupational/ Leisure-Time Physical Activity Separated in Final Analyses		Confounders Measured	Result	
Morabia, A *et al.* (2009) [26]	Assess the physical activity energy expenditure for transportation by car *vs.* subway *vs.* walking the same predetermined route.	Only the car *vs.* subway arms are relevant to this report.	Activity diary + GPS system used to calculate the average metabolic equivalent value for each study arm.	n/a as experiment limited to walking for transport.		no	Physical activity energy expenditure (Kcal/min) was significantly greater in subway users (2.35) compared to car users (1.74) travelling the same pre-determined route.	
USA								
Experimental 3 arm study								
N = 20								
Adults either working /studying at Queens College.								
Coogan, P *et al.* (2009) [27]	Association between neighbourhood urban form & physical activity.	(1) Shortest distance between each participants address & public transport.	Hours/week spent in utilitarian walking (≥5h walking/week *vs.* ¼ 5h walking/week).	Only measured utilitarian walking.		(1) Demographics, caregiver responsibilities, smoking/alcohol status, number of moves in last 2 years, energy intake, tv viewing, %vacant housing units, neighbourhood SES, crime index.	(1) Distance to transit (OR: 2.63 for lowest quintile distance to transit) & bus (OR: 3.23 for highest quintile bus routes) availability (when considered as only urban variable) is significantly positively associated with utilitarian walking.	
USA								
Longitudinal								
N = 20,354								
Black females 21–69 years old		(2) Bus availability (measured by miles of bus routes within 0.5 miles of individual’s address).				(2) As for 1+ adjusted for all other urban form variables (housing density, sidewalks, parks *etc* .).	(2) Bus availability is independently & significantly associated with utilitarian walking (OR 1.18–1.44 for lowest to highest quintile of bus route availability).	
Lindstrom, M (2008) [24]	Association between means of transport to work & overweight/ obesity	Means of transportation to work measured as options: walking, biking, car, bus	Not measured.	n/a as physical activity not measured		(1) Age, country of origin, education & time to travel to work.	(1) Odds of overweight + obesity(0.61–0.86) and obesity (0.51–0.95) in men who use public transport to travel to work are significantly lower compared with men who use a car to get to work.
Sweden							
Cross-sectional							
N = 16,705		train, other (could tick					
18–80 years old employed people		multiple)					
Brown, B *et al.* (2007) [28]	Does a new light rail stop increase number of light rail users & does light rail ridership relate to moderate activity bouts?	Pre-post building of new light rail stop.	Moderate intensity physical activity (MPA) measured with accelerometers & respondents indicated whether they were related to walking to/from light rail stop.	Respondents indicated whether the moderate intensity physical activity registered on accelerometers was associated with walking to/from light rail.		(1) Gender, household size & home ownership.	(1) Pre & post new stop rail rides were significantly related to more moderate intensity physical activity (MPA) bouts.
USA							
Pre/post test design							
n = 51							
Adults living within ½ mile of new rail stop						(2) #1 variables + moderate-intensity physical activity pre new rail stop.	(2) Longitudinal analysis showed walks to transit contributed to MPA above prior activity levels (before construction of new stop).
de Bourdeaudhuij, I *et al.* (2003) [39]	Examine the variance in sitting, walking and moderate- vigorous physical activity explained by a wide range of community design & recreational environmental variables above & beyond the variance accounted for by individual & group demographic variables.	Ease of access to public transport stop.	International physical activity questionnaire: minutes spent walking AND minutes spent in moderate-intensity physical activity (not walking) in last week.		no	(1) Sex, age, education, living situation, working situation, height, weight, body mass index.	(1) 3% of the variance in walking (all purposes) was explained by greater ease of walk to public transport (correlate = 0.16) and to land use mix.
Belgium							
Cross-sectional							
N = 521							
18–65 years old from Ghent							
Frank, L *et al.* (2010) [30]	Examine the relationship between energy used for active and motorised forms of transport & evaluate how modifiable features of the built environment are associated with the ratio between energy used for active *vs.* motorised travel.	-Distance to nearest public transport stop.	Average distance spent walking over 2 days (than converted to average kilocalories spent walking).		no	(1) Age, gender, ethnicity, drivers’ license status, income, #household members, vehicles in household.	(1) As shortest distance to nearest rail stop increased energy expended from walking decreased significantly
USA							
Cross-sectional							
N = 10,148		-“Transit				(2) As per #1.	(2) As shortest distance to nearest bus-stop increased, energy expended from walking increased significantly.
>16 years old residents of Atlanta		Accessibility” ( *i* *.e* *.* ,: whether respondent could access all the regions 5 major activity centers by walking to transit).				(3) As per #1.	(3) Those that had access to all 5 of the major city centres via transit burned significantly more kilocalories from walking.
Kamada, M *et al.* (2009) [40]	Describe environmental correlates (focus on public transport) of physical activity among rural Japanese women.	-Self report “access to public transport”.	International physical activity questionnaire used to assess time spent in occupational, leisure-time physical activity & transportation related walking over a typical 7 day week & divided into:recommendations)	no		(1) Age, body mass index, gender, general health state, household economy, employment, engagement in farming, parenting/care-giving status, driving status.	(1) “Sufficiently active” women significantly more likely (OR = 1.57) to report good access to public transport compared with inactive women.
Japan							
Cross-sectional		-GPS measured distance to train station.	(1) sufficiently active (meeting recommendations)				
N = 434		-Bus service convenience (combination of GPS measured distance to	(2) insufficiently active (not meeting				
40–64 years old rural Japanese women		bus-stop+bus frequency).	(3) inactive(no moderate-vigorous intensity physical activity)			(2) As per #1.	(2) Non-drivers in an area where bus services were moderately convenient were more likely (OR: 3.23) to be sufficiently active than those where it was less convenient.
Lachapelle, U *et al.* (2009) [31]	(1) Assess the relationship between using public transport & meeting recommended levels of physical activity while controlling for neighbourhood built environment & demographic factors.	-Distance to nearest transit stop/station.-	Average self-report distance walked for transportation over the 2 reporting days. 3 groups:	Only measured walking for transport		(1) Demographics, neighbourhood density, presence of services near work, distance from home to transit, car availability	(1) Only trips with public transport are significantly associated with being sufficiently active (OR: 3.35) compared to driving or being a car passenger.
USA							
Cross-sectional							
N = 4,156							
16–70 years old Atlanta residents	(2) Relationship between employer- sponsored public transport passes & walking.	If respondent received and used free/ subsidized transit pass.	-sufficient walking(meets recommendation)			(2) As per #1	(2) Having & making use of an employer-sponsored transit card positively & significantly associated with being sufficiently active (OR = 4.96) compared with not having access to a card.
			-moderate walking(less than sufficient but more than no walking)-no walking(no walking for transport)			(3) As per #1	(3) Transit users living 450–1,000 m of transit were significantly more likely to be moderate walkers (O = 6.54).
Li, F *et al.* (2008) [32]	Examine relationship between built environment factors, the prevalence of overweight/obesity & various forms of physical activity.	Density of public transport stations/stops.	-Walked for household errands/ transportation ≥30 min/week or not-Self-report moderate-vigorous intensity physical activity resulting in 3 categories: (1) met guidelines for moderate or vigorous physical activity.	yes		Age, gender, race/ethnicity, employment status, home ownership, income, health status, fruit & vegetable intake, fried food consumption, body mass index.	(1) Density of public transport stations significantly associated with more walking for transport & being “sufficiently active”.
USA							
Cross-sectional							
N = 1,221			(2) insufficiently active				
50–75 years old from 120 different neighbourhoods			(3) inactive			Residential density, median household income &% African American/Hispanic residents.	
Liao, Y *et al*. (2011) [33]	Examine the perceived environmental correlates of physical activity among normal weight & overweight Japanese men.	Access to public transport.	Categorised as either meeting the recommendations for walking and or moderate-vigorous intensity physical activity (excluding walking) or not meeting recommendations.	no		(1) Age, marital status, education, household income, employment status.	(1) Good access to public transport (OR = 2.3) is significantly associated with walking sufficiently per week to meet physical activity recommendations in normal weight men. This did not apply to overweight men.
Japan							
Cross-sectional							
N = 1,420							
30–59 years old Japanese men						(2) As per #1	(2) Good access to public transport had no significant relationship with moderate-vigorous intensity physical activity in normal weight & overweight men.
McConville, M *et al.* (2011) [34]	Association between accessibility/ intensity of non-residential land uses & walking for transport.	-Distance to bus-stop/ railway from person’s home measured	Walking for transport 3 categories:	Only measured walking for transport.		(1) Demographics +residential population density & sidewalk density.(2) As per #1(3) As per #1 + neighbourhood type	(1) Compared to not walking for transport the odds of walking for transport for ¼ 150 min/week were significantly lower with greater distance to bus stop (OR = 0.91, CI: 0.85–0.97).
USA							
Cross-sectional			(1) none				
N = 260			(2) ¼ 150 min/week				(2) Compared to not walking for transport odds for walking ≥150 min/week were significantly lower with greater distance to bus stop (OR = 0.01, CI:0.001–0.11) & rail station (OR = 0.9, CI:0.82–0.99).
Healthy adults from Montgomery County non-representative)		-# of bus-stops within ½ or ¼ mile.	(3) ≥150 min/week				(3) # of bus-stops within a ½ (OR:1.06) & ¼ (OR: 1.16) mile buffer associated with greater odds of walking ≥
							150 min/week for transport compared to not walking for transport.
Wilson, L *et al.* (2011) [6]	Examine how a range of objectively measured neighbourhood features are associated with likelihood of middle-aged adults walking in their local neighbourhoods.	Distance to bus-stop/ railway from person’s home measured.	Walking for leisure-time physical activity + transport walking 5 levels:	no		Demographics and neighbourhood level socioeconomic status and within neighbourhood variation in age, sex household type, education, occupation & household income.	(1) There was no relationship found between proximity to public transport & walking for any purpose (maybe because they didn’t specifically measure transport walking).
Australia (Brisbane)							
Cross-sectional			¼30 min				
N = 10,286			-≥30 min–¼90 min				
			->90 min–¼150 min				
			-≥150 min–¼300 min				
40–65 years old Brisbane residents from 200 neighbourhoods			-≥300 min				
Wen, L *et al.* (2008) [8]	Association between various modes of transport to work & overweight & obesity.	Whether public transport was usual method of commuting to work or not.	Not reported in terms of association with public transport (overweight/obese was instead).	Adjusted for people who met recommendedlevels of physical activity (all purpose).		(1) Age, marital status, education, language spoken at home, meeting recommendations for physical activity.	(1) Men who used public transport to get to work are significantly less likely to be overweight & obese (OR = 0.65; CI = 0.53–0.81) compared with men who drive to work (not significant for women).
Australia (NSW)							
Cross-sectional							
N = 6,810							
16+ years old working in NSW							
Pikora, T *et al.* (2006) [41]	Association between physical environmental factors & walking for recreation & transport near home.	Extent of presence of public transport within 400 m of home (embedded in a “destination score”).	Walking for transport near home or not in last 2 weeks (no time specified).	yes		(1) Demographics, socioeconomic status of area of residence + all other environmental variables (function, safety, aesthetic & destination).	(1) Presence of public transport 400m from home was not significantly associated with walking for transport (relationship was positive). However, the presence of destinations (including public transport) was significantly related to walking for transport near home (OR:1.8; CI: 1.33–2.44).
Australia (Perth)							
Cross-sectional							
N = 1,678							
18–59 years old Perth residents							
Lovasi, G *et al.* (2009) [42]	Test whether association between walkable environments & lower body mass index was stronger within disadvantaged groups.	-#bus/subway stops within 1 km radius of home.	Not measured (body mass index used)	n/a		(1) Age, gender, race, individual education, % of Black/Hispanic residents in area, % below poverty line.#1	(1) “Advantaged people” who have subway access & use public transport are significantly more likely to have a lower body mass index compared with disadvantaged groups.
USA							
Cross-sectional							
N = 13,102							
30+ years old New York residents		-Use public transport or not.				(2) As per	
Cerin, E *et al.* (2007) [43]	Examine the association of objective & perceived measures of access to destinations with self-reported walking for transport	-Proximity of public transport	Weekly minutes of transport-related walking	n/a as only measured transport-related walking		(1) Sociodemographics + neighbourhood selection (residents chose to live in area because of accessibility of certain destinations).	(1) No relationship between transport walking & proximity to public transport stops.
Australia (Adelaide)							
Cross-sectional							
N = 2,650							
20–65 years old recruited from 32 neighbourhoods in Adelaide		- Monthly frequency of walking to public transport.				(2) Sociodemographics and walking to specific types of destinations.	(2) Monthly frequencies of walking to public transport stop independently significantly associated with weekly minutes of walking for transport (b = 3.7).
